# Evaluation of cytochrome b sequence to identify *Leishmania* species and variants: the case of Panama

**DOI:** 10.1590/0074-02760200572

**Published:** 2021-04-19

**Authors:** Michelle Davila, Vanessa Pineda, José E Calzada, Azael Saldaña, Franklyn Samudio

**Affiliations:** 1 Universidad de Panamá, Facultad de Ciencias Naturales, Exactas y Tecnología, Panama, Panama; 2 Instituto Conmemorativo Gorgas de Estudios de la Salud, Laboratorio de Investigación en Parasitología, Panama, Panama; 3 Universidad de Panamá, Centro de Investigación y Diagnóstico de Enfermedades Parasitarias, Panama, Panama

**Keywords:** leishmaniasis, cytochrome b gene, haplotypes, Leishmania panamensis, Leishmania guyanensis, Panama

## Abstract

**BACKGROUND:**

The genetic heterogeneity of *Leishmania* parasites is a major factor responsible for the wide variety of *Leishmania*-associated manifestations. Consequently, understanding the genetic make-up of *Leishmania* species using suitable molecular markers is an important component of realising local and regional scale disease risk. The cytochrome b (cytb) is frequently used to type New World *Leishmania* species. However, its potential to discriminate *Leishmania* species and variants requires further evaluation.

**OBJECTIVES:**

To explore the capacity of cytb gene to identify New World *Leishmania* species and variants and to develop an approach able to type local *Leishmania* species and variants.

**METHODS:**

We retrieved 360 partial and complete *Leishmania* cytb gene sequences publicly available in GenBank database to study all single nucleotide polymorphisms (SNPs) across the cytb gene that differentiate New World *Leishmania* species. This information was used to develop an approach based upon the polymorphisms found in a DNA segment of 948bp. We also compared the typing results found with this technique with the polymerase chain reaction-restriction fragment length polymorphism (PCR-RFLP) profiling obtained using HSP70 gene as target. One hundred Panamanian isolates were used to both typed *Leishmania* species and assess local genetic variability.

**FINDINGS:**

We found complete agreement between our cytb approach and the PCR-RFLP profiling method based on HSP70 for *Leishmania* species identification. Ninety-two isolates were identified as *L. panamensis,* although other *Viannia* species were found circulating at a lower frequency. Three *L. panamensis* haplotypes were identified in Panamanian provinces. We also provide an initial report of *L. guyanensis* haplotypes circulating in Panama.

**MAIN CONCLUSIONS:**

Cytb gene sequence encompasses key main SNPs that aid to identify *Leishmania* species. The cytb approach developed with this information was able to identify and assess genetic variability of local *Leishmania* species found in this study.

Leishmaniasis, a neglected tropical disease transmitted by female sandflies and caused by kinetoplastic protozoa parasites of the genus *Leishmania*, is endemic in 98 countries worldwide. Due to the high number of cases and its geographical expansion, the World Health Organization (WHO) considers leishmaniasis as an emerging/reemerging vector borne parasitic disease.^1^ Regarding its impact on public health, leishmaniasis has an estimated annual incidence of 2.0 million cases and an approximated prevalence of 12,000,000 cases.^2^ In addition, this disease is responsible for 20,000 to 40,000 deaths occurring in rural and suburban populations all year round.^1^ ^,^ ^2^

Visceral and tegumentary leishmaniasis are the main clinical forms of the disease which show different clinical expressions depending upon the species of *Leishmania* responsible for the disease, the genetic background and immunological status of the host, and factors in the sand fly vector saliva.^3^ ^,^ ^4^ According to data reported by WHO in 2017, the cutaneous leishmaniasis is widely spread across the American continent and it is 15 times more frequent than the visceral form of leishmaniasis.^1^ After its first description in Panama more than one hundred years ago in 1910, leishmaniasis has now become the most prevalent vector-borne parasitic disease in the country with annual incidence as high as 3,000 new cases and afflicting people of all ages, but mainly those younger than 14 years old.^5^ ^,^ ^6^ ^,^ ^7^ Related to its neglected status, the transmission of cutaneous leishmaniasis in Panama is concentrated in sylvatic and rural areas affecting primarily socially and economically marginalised people who primarily present with cutaneous leishmaniasis (CL) and less than 3% with the mucocutaneous form (MCL).^1^

*Leishmania panamensis* is principal etiological agent of leishmaniasis in Panama as it has been found infecting anthropophilic vectors, mammalian reservoirs, and causing CL.^8^ ^,^ ^9^ ^,^ ^10^ However, other species of *Viannia* subgenera, such as *Leishmania braziliensis*, *Leishmania guyanensis* and *Leishmania naiffi* also circulate at a minor frequency in regions endemics for CL.^11^ Furthermore, an enzootic cycle of *Leishmania mexicana* and few human cases caused by this *Leishmania* species have been reported in Panama. In general, the evidence gathered so far, indicates that *Leishmania Viannia* species are more prevalent in Panamanian endemic areas.^8^ ^,^ ^10^ ^,^ ^12^ ^,^ ^13^

The genetic make-up of *Leishmania* defines biological traits responsible for parasite survival in mammals and vector hosts and its capacity to evoke a particular clinical form. The broad spectrum of *Leishmania*-associated symptoms described in an endemic area are determined by both parasite and hosts’ genetic composition. On the other hand, clonal diversity, and genetic heterogeneity responsible for variability in parasite virulence are quite common in *Leishmania.* ^14^ ^,^ ^15^ Thus, it is important to gather information on the genetic background of *Leishmania* species circulating in specific endemic areas to strengthen local epidemiological surveillance programs and provide proper disease treatment.

*L. panamensis* has been associated to diverse cutaneous forms of the disease, a behavior that might be the result of local interplay between *L. panamensis* variants and human populations with different genetic backgrounds.^16^ ^,^ ^17^ ^,^ ^18^ ^,^ ^19^ Little is known about the population structure of *L. panamensis* in Panama. Local studies focus mainly on discriminating *Leishmania* species on vectors, hosts, and humans rather than inferring their genetic diversity.^10^ ^,^ ^20^ ^,^ ^21^ Therefore, it is important to conduct further studies in our country using molecular markers that can differentiate *Leishmania* at the subspecies level. Since haplotypes might arise as an intrinsic attribute of population genetic variation, it is feasible to use these targets as molecular markers in studies aiming to assess the genetic structure of a particular etiological agent.^22^ As a result, it is worth to explore the potential of molecular targets for uncovering different haplotypes that could be circulating in different *Leishmania* transmission cycles. In this regard, some molecular approaches based on cytb gene have already been used in some Latin American countries to discriminate species and haplotypes of *Leishmania*.^23^ ^,^ ^24^ ^,^ ^25^ However, most of these studies were based on partial cytb sequences and no description of the polymorphic sites used to discriminate *Leishmania* species and variants were reported. Thus, it might be necessary a further evaluation of the cytb gene-based methods or develop new ones assessing the extent of polymorphic sites and their capacity to type species and variants of *Leishmania*. In this sense, we developed a molecular approach based upon the polymerase chain reaction (PCR)-amplification and sequencing of a 948 bp segment of cytb gene that showed enough single nucleotide polymorphisms (SNPs) to discriminate New World *Leishmania* species. Also, to shed light on the local genetic variability of *Leishmania*, we evaluated one-hundred isolates from eastern and western side of Panama.

## MATERIALS AND METHODS

Design of the PCR approach

*In silico design of the PCR approach* - We developed a PCR approach able to discriminate new world *Leishmania* species based on the amplification by PCR and Sanger-sequencing of a 948 bp cyt b gene sequence. To develop this PCR-approach, we retrieved 360 partial and complete *Leishmania* cytochrome b (cytb) gene sequences publicly available in GenBank database including: *L. braziliensis* (110), *L. peruviania* (29), *L. guyanensis* (80), *L. panamensis* (6), *L. lainsoni* (22), *L. naiffi* (5), *L. shawi* (3), *L. mexicana* (39), *L. amazonensis* (17), *L. infantum/L. donovani* (39), *L. tarentolae* (1), *L. major*(1), *L. aethiopica* (1), *L. tropica* (3), *L. aristidesi* (1), *L. garnhami* (1) and *L. pifanoi* (1). Twenty-eight out of the 360 cytb sequences corresponding to the pre-edited version of cytb gene were used to design specific primers (Table I). The rest of the sequences allowed us to analyse polymorphic sites [Supplementary data (Table I)]. All sequences were downloaded to the bioinformatic software UGENE v.34 version and were aligned using the MAFFT algorithm included on the software.^26^ Sequence alignment was used to find consensus regions for all *Leishmania* species which were then employed to design five primers sets using the algorithm of primer3 incorporated in UGENE. Important characteristic of primers sets, like formation of secondary structures and hairpins was evaluated *in silico* by OligoAnalyzer tool (Integrated DNA technology) and their specificity was assessed using primer-Blast tool (NCBI).

**TABLE I:** Complete cytochrome b (cytb) sequences retrieved from GenBank to design *Leishmania* specific primers

Length	GenBank accession number	*Leishmania* specie
1398pb	M10126.1	*Leishmania tarentolae*
1078pb	BK010875.1	*Leishmania panamensis*
1078pb	AB095968.1	*Leishmania panamensis*
1078pb	MK570510.1	*Leishmania panamensis*
1078pb	AB434682.1	*Leishmania braziliensis*
1078pb	AB434681.1^*^	*Leishmania braziliensis*
1078pb	AB095967.1^*^	*Leishmania braziliensis*
1078pb	AB095966.1	*Leishmania braziliensis*
1078pb	AB095969.1	*Leishmania guyanensis*
1078pb	BK010883.1	*Leishmania shawi*
1078pb	AB434680.1	*Leishmania shawi*
1079pb	AB095963.1^*^	*Leishmania mexicana*
1078pb	AB095964.1	*Leishmania amazonensis*
1079pb	AB434679.1	*Leishmania pifanoi*
1079pb	AB095965.1	*Leishmania garnhami*
1078pb	AB434678.1	*Leishmania aristidesi*
1079PB	HQ908261.1^*^	*Leishmania infantum*
1079pb	HQ908267.1^*^	*Leishmania donovani*
1079pb	HQ908262.1^*^	*Leishmania donovani*
1079pb	AB095957.1	*Leishmania donovani*
1079pb	AB095959.1^*^	*Leishmania infantum*
1079pb	AB095958.1	*Leishmania infantum*
1079pb	AB434677.1	*Leishmania archibaldi*
1080pb	AB095961.1	*Leishmania major*
1080pb	AB095962.1	*Leishmania aethiopica*
1080pb	AB095960.1	*Leishmania tropica*
1080pb	HQ9082270.1	*Leishmania tropica*
1080pb	HQ9082270.1	*Leishmania tropica*
1080pb	HQ908257.1	*Leishmania tropica*

*: reference *Leishmania* sequences used for the phylogenetic analysis.

The best set of primers based on the characteristics evaluated were cytb-VF 5’-AAGCGGAGAGAAAAGAAAAGG-3’ and cytb-VR 5’-AATGAATAAGTAAATCATAATAAC-3’ which align to regions 1-21 and 925-948 of cytb gene, respectively, and amplify a PCR-product of 948 bp. This set of primers was then employed to standardise a PCR-sequencing approach aiming to identify *Leishmania* species and cytb haplotypes.

*Average of genetic distances between groups of cytb sequences* - The average of genetic distances between nine groups of *Leishmania* species depicted on Table II were inferred by Mega X software using cytb gene reference sequences depicted on Table I.^27^ The rate variation among sites was modeled with a gamma distribution (shape parameter = 4) and all positions including coding and noncoding ones were included in the analysis. All ambiguous positions were removed for each sequence pair.

**TABLE II:** Estimates of evolutionary divergency over sequence pairs between groups of *Leishmania* cytochrome b (cytb) sequences

	1	2	3	4	5	6	7	8	9
1. *L. tarentolae*									
2. *L. panamensis*	0.10								
3. *L. braziliensis*	0.10	0.01							
4. *L. guyanensis*	0.10	0.01	0.01						
5. *L. shawi*	0.10	0.01	0.01	0.01					
6. *L. mexicana complex*	0.11	0.12	0.12	0.12	0.11				
7. *L. donovani complex*	0.12	0.12	0.11	0.11	0.11	0.10			
8. *L. major*	0.13	0.13	0.12	0.13	0.12	0.11	0.08		
9. *L. tropica complex*	0.13	0.13	0.12	0.13	0.12	0.10	0.08	0.07	

*Ethical statement* - This research was considered by the Comité de Bioética de la Investigación del Instituto Conmemorativo Gorgas de Estudios de la Salud (CBI-ICGES), and deemed exempt (Note No. 1084/CBI/ICGES/16).

Evaluation of the PCR approach

**Fig. 1:**
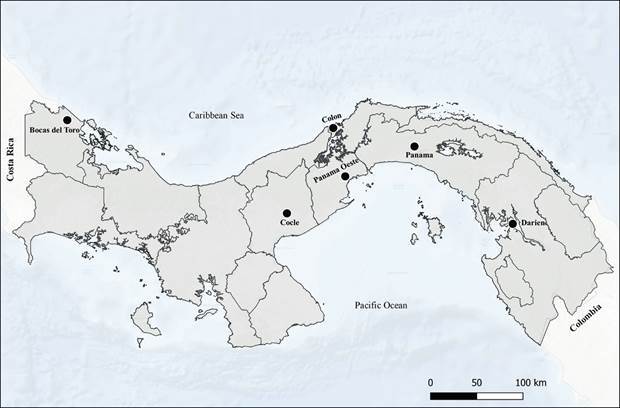
geographical positions of the Panamanian Provinces where *Leishmania* spp. isolates were obtained.

*Isolates* - One hundred *Leishmania* isolates from both eastern border and western borders of Panama Canal were used in this study (Fig. 1). Isolates were collected during the last five years (2015-2020) and stored in liquid nitrogen at the Departamento de Investigación en Parasitología, Instituto Conmemorativo Gorgas de Estudios de la salud (ICGES) situated in Panama City. Parasites were isolated from patients living in Provinces located east (Panama and Darien Provinces) and west (Colon, Panama Oeste, Cocle and Bocas del Toro) of the Panama Canal (Fig. 1). These locations are recognised endemic areas of leishmaniasis in the country with a long-term average of CL incidence rates between 5-25 new cases per 10,000.^28^

*Parasite culture and DNA extraction* - *Leishmania* stocks were cultured using Schneider’s insect medium (Sigma Aldrich, Inc., St. Louis, USA) supplemented with 20% heat-inactivated fetal bovine serum (Gibco, Grand Island, USA) at 26ºC. Total genomic DNA was extracted from promastigotes using a commercial kit (Wizard Genomic DNA Purification Kit, Promega, Madison, WI) according to the manufacturer’s instructions.

*PCR conditions* - The optimal conditions for amplifying the selected cytb gene fragment consisted of the addition of one unit of Platinum high fidelity taq polymerase, 0.2 μM of dNTs, 2.5 mM of MgSO_4_, 0.3 μM of primers forward and reverse, 2.5 μL of 10X high fidelity buffer, 50 ng of genomic DNA and water up to a final volume of 25 μL. Target DNA was amplified after a primary denaturation of 95ºC for 5 min following 35 cycles consisting of 96ºC for 10 s, 50ºC for 30 s and 68ºC for 60 s and a final extension of 68ºC for 10 min.

*Cytb gene sequencing and phylogenetic analysis* - The cytb amplification products were run in 1.2% agarose gels prepared with 0.5X TBE buffer and stained with GelRed® (Biotium). PCR products were cleaned up by PureLink^TM^ PCR purification kit (Invitrogen) following manufacturer’s instructions. DNA sequencing of both strands was carried out using PCR-primers abovementioned and BigDye Terminator 3.1 cycle sequencing kit (Applied Biosystems). Primers and deoxynucleotide triphosphates were removed using Big dye Xterminator^TM^ (Applied Biosystems) and sequencing reactions were run on ABI 3500XL sequencer. Sequencing chromatograms were edited using the assembling-to-reference tool of UGENE toolkit using a trimming quality value of 25. After edition, a segment of 792 bp of cytb sequence were used to perform the phylogenetic analysis. Sequences were then multiple aligned using the MAFFT algorithm also included in UGENE with a maximum number of iterative refinements of 3 and a gap penalty of 1.53. The HKY (G + I) model was found as the best DNA evolution model by the software JModelTest 2.^29^ A phylogenetic tree reconstruction of *Leishmania* was implemented applying Bayesian inference (BI) with the Mr. Bayes v.3.2 software.^30^ Ten Markov chains were proceeded for ten million of generations, and trees were sampled for every 7,000 generations setting the program to run the substitution model with the option invgamma and permit different rates of transition and transversion (K80 or HKY85 model). Twenty-five percent of the sampled trees were discarded, and the remaining were used to build up a consensus tree and to calculate posterior probabilities of clades. The result of Bayesian analyses was visualised using Figtree v1.4.2. The cytb gene sequence of *Leishmania siamensis* (JX195634.1) was used as outgroup. Cytb gene reference sequences used to perform this analysis are showed in Table I and Supplementary data (Table I) with the GenBank accession number marked by an asterisk. Transformation of the leaves and a schematic representation of the root were applied for visualisation purposes.

*HSP70-RFLP analysis* - Eighty isolates of *Leishmania* spp. were used to typed *Leishmania* species by PCR-RFLP approach based on HSP70 gene. PCR was performed with oligonucleotides F25 and R1310, that amplify a 1286 bp product from the repeated gene heat shock protein 70 (hsp70) as previously described.^31^ Amplification reactions were performed in a final volume of 50 μL containing 25 ***μ*** L of Go Taq Green Master Mix 2X (Promega), 0.6 μmol/L of each primer and 5 μL of DNA. The resulted amplicons were digested with *Hae* III and subsequently with *BccI* or *Rsa*I endonucleases. Obtained restriction patterns were analysed by electrophoresis and compared with reference strain patterns.

*Haplotype analyses* - The sequences recognised as haplotypes herein, were identified from aligned cytb sequences of *L. panamensis* displayed in UGENE Alignment Editor. Sequences from identified haplotypes were then evaluated in PopART software v.1.7 to construct a median joining network among haplotypes.^32^

## RESULTS

*Evaluation of partial and complete cytb sequences* - Fifteen percent (15%) of the evaluated 948 bp cytb sequences consist of 145 main polymorphic sites, including 42 singletons and 103 parsimony-informative sites that can discriminate members of the *L. Viannia* subgenus, species of *L. mexicana* complex and distinguish the three complexes of New World *Leishmania* [Supplementary data (Table II)]. Furthermore, this cytb sequence encompassed 35 polymorphic specific sites for species of subgenus *Viannia*, 15 SNPs that differentiate *L. mexicana* from *L. mexicana amazonensis*, 50 SNPs that aid to discriminate members of *L. mexicana* complex from subgenus *Viannia* complexes, and 24 singletons that are found in *L. infantum* as well as *L. donovani*.

Table II shows estimates of evolutionary divergency over sequence pairs between groups consisting of the pre-edited version of cytb sequences from nine groups of *Leishmania* species. A low rate of divergency was found in *Leishmania* species of the subgenus *Viannia* as the total genetic distance between groups of them was 0.01. However, we found enough SNPs across the 948 bp cytb sequences to discriminate species of this subgenus except for *L. peruviania* and *L. braziliensis* which had great similarity in nucleotide sequence. As we analysed partial cytb sequences of *L. peruviana* starting from position 74 onwards, we do not rule out the presence of any SNPs that permit to discriminate these species somewhere upward this position. Conversely, we found high genetic distances ranging from 0.11 to 0.13 between cytb sequences from *Viannia* subgenus and grouped cytb sequences belonging to *L. mexicana* complex, *L. donovani* complex, *L. tropica* complex, *L. major* and *L. tarentolae*. This finding demonstrates that the cytb gene segment selected for our PCR-approach is useful to distinguish species belonging to the different *Leishmania* complexes.

**Fig. 2:**
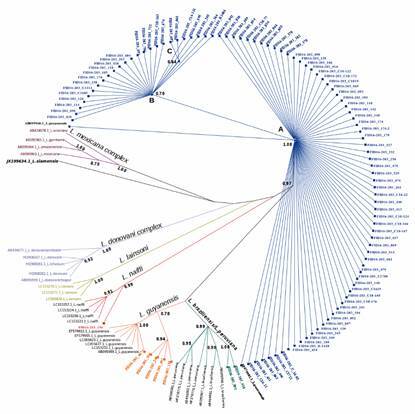
phylogenetic tree inferred from Bayesian analysis of cytochrome b (cytb) gene using Mr. Bayes v. 3.2. The numbers at the node represent Bayesian posterior probabilities; *Leishmania siamensis* JX195634.1 was used as the outgroup. Different labels colours in *L. panamensis* (blue), *L. guyanensis* (orange), *L. braziliensis* (green) and *L. naiffi* (red) phylogenetic groups represent isolates of these species found herein. The letters A, B and C on the tree depict *L. panamensis* haplotypes.

*Phylogenetic inference* - The phylogenetic inference using the cyt b sequences clearly separated and grouped in clusters *Leishmania* species from *L. mexicana* complex, *L. donovani* complex and subgenus *Viannia* (Fig. 2). All local isolates analysed in this study were found to belong to subgenus *Viannia*. The partial cytb gene sequences obtained from these isolates were deposited in GenBank under accession numbers MW117326 to MW117425. Ninety-two percent of all field isolates clustered with *L. panamensis* reference sequences, branching into two major groups that include three *L. panamensis* haplotypes. The remaining 8% of the field isolates were identified as *L. guyanensis*, *L. braziliensis*/*L. peruviana* and *L. naiffi*, according to the phylogenetic analysis (Fig. 2). As for *L. guyanensis* local isolates, one of them clustered with *L. guyanensis* reference group and the rest of the isolates clustered into a sister subclade laying close to this group on the tree. The presence of two *L. guyanensis* different subclades after the phylogenetic analysis indicates the circulation of at least two different haplotypes of this *Leishmania* specie in Panama. On the other hand, local *L. braziliensis* and *L. naiffi* isolates clustered with their respective reference groups conformed by cytb gene sequences retrieved from GenBank.

*PCR-RFLP analysis of HSP70 gene* - We found 100% of concordance between cytb approach described here and the PCR-RFLP based on HSP70 when used to identify eighty *Leishmania* isolates from different regions of Panama (Table III). All isolates considered by cytb approach as *L. braziliensis / L. peruviana* were confirmed by the HSP70 based approach as *L. braziliensis* isolates.

**TABLE III:** *Leishmania* identification by polymerase chain reaction-cytochrome b (PCR-cytb) gene sequencing and PCR-restriction fragment length polymorphism (PCR-RFLP) based on HSP70 gene

*Leishmania Viannia* species	Cyt b approach (n = 80)	Hsp70 approach (n = 80)
*L. panamensis*	72	72
*L. braziliensis*	5^*^	5
*L. guyanensis*	2	2
*L. naifﬁ*	1	1

*: isolates identified as *L. braziliensis* after confirmation by PCR-RFLP approach based on HSP70 gene.

**Fig. 3:**
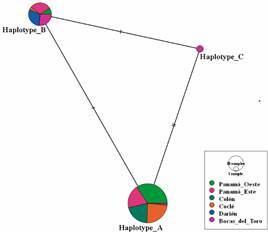
haplotype network inferred by a median-joining method using cytochrome b (cytb) sequences from *Leishmania panamensis*. Circles represent different haplotypes. Circle size is proportional to haplotype frequency; colours indicate geographical origins. Hatch marks correspond to nucleotide substitutions.

*Leishmania haplotypes* - We found three cytb haplotypes of *L. panamensis* circulating in the studied areas that were assigned as haplotype A, haplotype B, and haplotype C as depicted on Fig. 3. Only two non-synonymous SNPs at positions 357 and 669 of the *L. panamensis* cytb gene complete sequence distinguished these haplotypes. One of them is a transition C↔T at position 357, where haplotype B and C showed the nucleotide cytosine and the haplotype A thymine instead. Additionally, at position 669 of cytb sequence we found an A↔G transition. In this nucleotide position, haplotypes A and B showed the base guanine and the haplotype C adenine instead. As a result, two SNPs distinguish haplotype A from C and only one SNP differentiate haplotype A from B and haplotype B from C.

The number of *L. panamensis* haplotypes found by province is shown in Table IV. All *L. panamensis* isolates (n = 92) were used to identify haplotypes from provinces located to the west and east side of the Panama Canal. Three haplotypes of *L. panamensis* were found distributed across the studied provinces (Fig. 3). The most frequent haplotype (A) was found in 67 out of 92 *L. panamensis* cytb sequences and occurred in almost all evaluated provinces, except in Darien where only haplotype B was identified. Conversely, haplotypes B and C seem to circulate at a lower frequency since only 25 and 2 cytb sequences from both haplotypes, respectively, were detected in this study. Haplotype B was found in all provinces except for Colon, and haplotype C was only observed in Bocas del Toro Province. However, because a low number of isolates from Bocas del Toro (9) and Darien (7) were evaluated, we do not rule out the circulation of other haplotypes at frequencies different from the ones described in this study.

**TABLE IV:** Total number of *Leishmania panamensis* haplotypes found in the study by localities

Localities	Haplotype A	Haplotype B	Haplotype C	Haplotypes found by province
Panama Oeste	23	2	0	25
Colón	14	0	0	14
Bocas del Toro	1	6	2	9
Coclé	16	1	0	17
Panamá Este	13	7	0	20
Darién	0	7	0	7
Total number of haplotypes	67	23	2	92

Two *L. guyanensis* haplotypes were found circulating in Cocle and Panama Oeste province in this study. Remarkably, these haplotypes showed greater diversity than *L. panamensis* haplotypes as we found six polymorphic sites at position 119 (T↔C), 241 (G↔T), 313 (A↔T), 619 (G↔A), 686 (A↔G) and 860 (C↔T). Only one SNP at position 686 was nonsynonymous causing the amino acid replacement isoleucine to valine in cytb protein. Since only five isolates of *L. guyanensis* were detected in this study, there is a high probability that other *L. guyanensis* haplotypes might be circulating in these endemic areas.

## DISCUSSION

Cytb is one of the set of cytochromes responsible for the electron transport process initially used for the phylogenetic analysis of *Leishmania* genus.^33^ As molecular marker for *Leishmania* spp., cytb is a good typing option providing its good resolution for all tested species across the globe. It is also sensitive enough for use on clinical and environmental samples, and it is readily analysed.^34^ However, to increase its reliability, it is important to validate its performance using a more extended set of sequences from strains belonging to current *Leishmania* complexes and from different geographical regions, including parasites isolated from reservoirs and vectors.

The availability of *Leishmania* genomic sequences increases as more studies are carried out using specific molecular markers or even complete genomes. This the case of cytb gene, which has been utilised successfully to discriminated new world and old world *Leishmania* species and to assess their populational structure, a fact that has increased the number of cytb sequences publicly available in databases.^25^ ^,^ ^33^ ^,^ ^35^ ^,^ ^36^ Herein, we describe 145 main SNPs found across the 948 bp segment selected of the cytb gene after evaluating 360 complete and partial sequences from New World *Leishmania* species belonging to different complexes. Altogether, these SNPs are responsible for the genetic divergence seen between *L. Viannia* species and species from *L. mexicana* and *L. donovani* complexes, particularly considering that no size differences were observed between complete cytb sequences of these species. The first report of cytb gene variation in *Leishmania* evaluated 15 strains, and found 245 polymorphic sites and an average sequence identity between species from *Leishmania* complexes of 90%.^35^ In an additional study by these authors, 30 additional strains of *Leishmania* were evaluated highlighting the usefulness of cytb for phylogenetic analysis. In that study, Lugo and collaborators were able to group *Leishmania* cytb sequences in five main complexes using completes cytb sequences.^33^

Our results suggest that the 948bp segment contains 60% of the polymorphic sites described across the cytb gene in the first report, exhibiting enough sequence divergence to discriminate species from New World *Leishmania* complexes. Moreover, its usefulness is supported by the availability of a larger set of *Leishmania* sequences and the presence of an adequate amount of SNPs as source of phylogenetical signal necessary to discriminate *Leishmania* species as reported elsewhereusing completes cytb gene sequences.^33^ As depicted in Fig. 2, singletons and parsimony informative sites across the 948 bp cytb segment allowed a clear separation of *L. Viannia* species from species of both *L. donovani* and *L. mexicana* complexes. Nonetheless, our phylogenetic approach based upon the 948 bp cytb sequence did not separate *L. donovani* and *L. infantum* nor *L. braziliensis* and *L. peruviana*. The same results were described using a phylogenetical analysis based upon the complete cytb sequence.^34^ The species status of *L. peruviana* within the *L. braziliensis* complex is a current matter of discussion. However, a comparative genomic analysis of both species found interspecific SNP/Indel differences and chromosome copy number variations that support that they are closely related but distinct species.^37^ As approaches based on HSP70 gene or Multilocus typing are able to differentiate both species, any of these tools could be used in conjunction with cytb to confirm the circulation of these species and identify others.^31^ ^,^ ^38^ Alternatively, methods based on PCR and sequencing of genes HSP70, miniexon and ITS1can differentiate *L. donovani* complexes, making it easy to use in combination with the cytb gene as complementary tool in places where tegumentary and visceral leishmaniasis overlapped.^39^ Nonetheless, additional set of markers with a better discriminatory power should be applied to clarify any nuclear-mitochondrial disagreement, a fact that already has been reported in *Leishmania* typing.^25^ Also, our PCR approach was designed to perform molecular typing of *Leishmania* isolates using a known quantity of genomic DNA, a fact that might affect the sensitivity of this methodology if used with clinical samples because of the low-copy number of cytb gene.

We found a high grade of agreement between the cytb approach developed in this study and the one based on PCR/RFLP of HSP70 gene when typing Panamanian isolates using both techniques. Indeed, the PCR-RFLP analysis of HSP70 gene aid to confirmed five isolates typed as *L. braziliensis*/*L. peruviana* by cytb approach as *L. braziliensis*. This fact points out that our cytb approach has enough discriminatory power to discriminate *L. Viannia* species that it is the case of the ones causing CL in Panama. However, some cases of disagreement have been reported when used HSP70 based molecular tools and approaches based on mitochondrial gene to identify New World *Leishmania* species.^25^ In spite of this divergence, we believe that the use of the approach presented herein in combination with HSP70 based molecular tools might improve *Leishmania* species assignment as showed in different *Leishmania* typing studies.^25^ ^,^ ^40^

*L. panamensis* was the predominate species isolated throughout all geographic regions studied in Panama (Fig. 2). Previous studies carried out in Panama support the wide distribution and prevalence of human cases of CL caused by *L. panamensis.* ^9^ ^,^ ^10^ Besides, this species has been found naturally infecting all anthropophilic sand flies,^41^ and it is also responsible for the high infection rates found in the two-toed sloth; a major sylvatic reservoir widely distributed in *Leishmania* endemic areas of Panama.^8^ ^,^ ^42^ The rest of the isolates from this study belongs to *L. guyanensis* (5), *L. braziliensis* (2) and *L. naiffi* (1). These species have been recently reported circulating at low frequencies in the country.^11^ This fact has important epidemiological implications for the local surveillance and control programs as well as for the clinical management of patients. In this line, it would be necessary initiatives aiming to monitor and determine geographical distribution, dynamic of infections, frequency of circulation of new reported species and to establish their transmission cycles. From clinical point of view, disease outcome and treatment prognosis are influenced by *Leishmania* species.^43^ In this context, *L. braziliensis* and *L. guyanensis* have been associated with localised cutaneous leishmaniasis, disseminated cutaneous leishmaniasis and mucosal lesions.^44^ In the case of *L. naiffi*, no association between this species and mucosal leishmaniasis has been observed. Moreover, cutaneous lesions occasioned by *L. naiffi* are single, smalls and located on hands, arms or legs.^45^ On the other hand, *L. braziliensis* isolates with reduced drug uptake have been recovered from atypical lesions, and some mutant variants of *L. guyanensis* strains are resistant to pentavalent antimonials, the first-line treatment option for CL in Panama.^46^ ^,^ ^47^ Interestingly, a poor response to antimonial or pentamidine therapy has already been described in patients infected with *L. naiffi.* ^48^ Thus, the sympatric circulation of different *Leishmania* species from subgenus *Viannia* in Panama should be further studied because it might have significance regarding human disease pathogenesis and control.

Three *L. panamensis* haplotypes were found circulating in the study areas (Fig. 3). A previous study conducted in Colombia using a partial sequence of cytb as a barcode, analysed 201 *L. panamensis* cytb sequences. A great variability was found supported by the existence of 80 SNPs across the cytb sequence.^23^ Herein, we only identified three SNPs along the *L. panamensis* cytb sequence responsible for the haplotype assignments. This might reflect a low genetic diversity of *L. panamensis* due to the expansion and fitness of few variants in both anthropophilic vectors and human beings. In this line, a pilot study conducted in Morocco using ITS1 as marker suggested that the overrepresentation of one haplotype over the rest in an endemic focus might be result of this fitness.^49^

It seems that there are differences in abundance and frequency of *L. panamensis* haplotypes in the studied sites. Haplotype A predominated in the studied sites as it was found in almost all evaluated Provinces except for Darien. Conversely, haplotype B showed a high frequency in Bocas del Toro and Darien Provinces, and only two cytb sequences belonging to Haplotype C were found in Bocas del Toro. Apparently, there is an association between genetic diversity and geographical origins of the *L. panamensis* isolates. In this sense, haplotype A might be involved in most of the current leishmaniasis transmission cycles in Panama along with local anthropophilic species of *Lutzomyia* and *Choleopus hoffmani*. While haplotype B and C might be circulating only in sylvatic transmission cycles, where human settlements are surrounded by tropical rain forest at the human-forest interface. To confirm this finding, further studies are necessary considering all components in the transmission cycles in which *L. panamensis* haplotypes participate. It is also possible that the low number of isolates evaluated from some Provinces have led to a sample bias that keep us from suggesting the overall predominance of haplotype A or even ruling out the circulation of other haplotypes.

The transmission cycle of *L. guyanensis* has been mainly associated with the presence of human beings in forest regions.^47^ In this sylvatic environment, animals such as sloths, anteater, marsupials and rodents are responsible for maintaining parasite populations.^50^ Two *L. guyanensis* haplotypes were found circulating in communities located through the central mountain range of Panama specifically in Cocle and the mountainous regions of Panama Oeste. These regions are considered wet/rain environments with a high proportion of forest coverage and where CL shows a high incidence.^28^ In these regions, as other endemic regions of the country, a part of the original forest environment has been preserved, with an abundance of anthropophilic sand flies and the presence of several *Leishmania* reservoirs, with a high rate of human infection.^41^ Therefore, it is feasible that under this eco-epidemiological scenario a zoonotic cycle has been established in which different lineages of *L. guyanensis* are participating. In this context, our results suggest the circulation of two variants of *L. guyanensis* that somehow might be associated with transmission cycles of CL in Coclé and Panama Oeste Provinces. This *Leishmania* species displays great genetic plasticity due to a high level of sexual recombination in vectors, complemented with a clonal expansion in natural hosts which are the main responsible for its diffusion in endemic regions.^51^ Thus, sylvatic cycles currently established in Cocle and Panama Oeste combined with a high abundance and diversity of parasite vectors and reservoirs meet all necessary characteristics for the emergency and dissemination of different *L. guyanensis* genotypes. However, entomological, and zoological data are required to support this hypothesis.

Using ribosomal fingerprinting, Rotureau et al. observed two different non sympatric lineages of *L. guyanensis* in French Guiana associated with regions characterised by dense primary rain forest spotted with gold-digging sites where anthropogenic changes were occurring.^52^ In Panama, opencast copper mining activities in Cocle and Colon Provinces has led to major man-made environmental changes including boosting human settlements and human exposure to already established zoonotic cycles of *Leishmania* parasites. Therefore, human infections with different *L. guyanensis* genotypes already circulating in these areas are plausible and might become a public health concern in these regions of Panama.

The genetic background of *Leishmania* species is thought to be one of the main factors influencing leishmaniasis clinical outcomes. Therefore, it is important to associate existent clinical effects with genetic variants of *Leishmania* spp. circulating in different epidemiological scenarios using appropriate molecular markers. There is limited data regarding the genetic variation of *Leishmania* spp. based on the sequencing of cytb gene. A study carried out in southern Iran found that typical and atypical cutaneous lesions were caused by different *Leishmania major* variants.^53^ Approaches based on the sequencing of cytb gene have been used mainly to identify New World *Leishmania* species without making an association with clinical outcomes.^23^ ^,^ ^25^ ^,^ ^54^ ^,^ ^55^ As the goal of our study was to assess the potential of cytb gene sequence to discriminate *Leishmania* species and variants, we did not look for an association between the haplotypes found herein and the clinical manifestations seen in Panamanian patients. However, we do not rule out that this association might exist as one approach based on HSP70 found a strong correlation between genetic variant of *L. braziliensis* and different clinical manifestations.^56^

Altogether, our results highlight the presence of different *L. Viannia* species and genetic variants within *L. panamensis* and *L. guyanensis* circulating in the country. The plethora of clinical characteristics seen in the country might be the result of infections with these species or their variants. To evaluate possible associations between genetic variants and clinical expression of the disease, it is necessary to use suitable molecular markers. Sequence variations of the cytb gene have already been related with different CL clinical outcomes.^43^ Consequently, an approach like the one presented in this article based on cytb gene that shows enough SNPs to identify New World *Leishmania* species and their variants represent a good option for *Leishmania* species identification in Latin American countries afflicted by leishmaniasis.
