# Supplementary material for: Evaluation of cytochrome b sequence to identify Leishmania species and variants: the case of Panama
Source: Mem Inst Oswaldo Cruz. 2021 Apr 19;116:e200572. doi: 10.1590/0074-02760200572 (PMC8061342; doi:10.1590/0074-02760200572)
Supplement: Supplementary file 1 [file 1678-8060-mioc-116-e200572-s.pdf]

TABLE I

Partial cytochrome b (cytb) sequences retrieved from GenBank to describe polymorphic sites that discriminate *Leishmania* complexes

Length	GenBank accession number	*Leishmani*a specie	Isolate	Country of isolation
872bp	EF579900.1	*Leishmania braziliensis*	LEM2252	Brazil
817bp	LC153268.1	*Leishmania braziliensis*	*15-10DA1*	Ecuador
817bp	LC153255.1	*Leishmania braziliensis*	14-2CBM2	Ecuador
817bp	LC153229.1	*Leishmania braziliensis*	13-9CBM1	Ecuador
817bp	LC153194.1	*Leishmania braziliensis*	12PVM15	Ecuador
857bp	MF278774.1	*Leishmania braziliensis*	MHOM/PE/84/LC53	Peru
730bp	MF344881.1	*Leishmania braziliensis*	EM_099	Brazil
730bp	MF344880.1	*Leishmania braziliensis*	EM_062	Brazil
730bp	MF344879.1	*Leishmania braziliensis*	EM_255	Brazil
730bp	MF344878.1	*Leishmania braziliensis*	EM_244	Brazil
730bp	MF344877.1	*Leishmania braziliensis*	EM_165	Brazil
730bp	MF344876.1	*Leishmania braziliensis*	EM_154	Brazil
730bp	MF344875.1	*Leishmania braziliensis*	EM_078	Brazil
817bp	LC055635.1	*Leishmania braziliensis*	13AM5	Ecuador
817bp	LC055633.1	*Leishmania braziliensis*	13AM1	Ecuador
817bp	MH841942.1	*Leishmania braziliensis*	AB2	Argentina
817bp	MH841941.1	*Leishmania braziliensis*	AB1	Argentina
713bp	LC472875.1	*Leishmania braziliensis*	12-2PI3	Peru
706bp	LC472870.1	*Leishmania braziliensis*	12-2AA4	Peru
754bp	LC472868.1	*Leishmania braziliensis*	12-2AA2	Peru
795bp	LC472866.1	*Leishmania braziliensis*	16-1MD-L301	Peru
773bp	LC472865.1	*Leishmania braziliensis*	17-11HU1	Peru
696bp	LC472864.1	*Leishmania braziliensis*	17-11MD13	Peru
697bp	LC472861.1	*Leishmania braziliensis*	12-2PP9	Peru
714bp	LC472860.1	*Leishmania braziliensis*	12-2PP8	Peru
714bp	LC472855.1	*Leishmania braziliensis*	12-2PP3	Peru
716bp	LC472854.1	*Leishmania braziliensis*	12-2PP2	Peru
714bp	LC472847.1	*Leishmania braziliensis*	12-2San3	Peru
817bp	LC472485.1	*Leishmania braziliensis*	16-12MD-CL2	Peru
817bp	LC472484.1	*Leishmania braziliensis*	16-12MD-CL1	Peru
817bp	LC472481.1	*Leishmania braziliensis*	16-1MD79	Peru
817bp	LC472483.1	*Leishmania braziliensis*	16-1MD68	Peru
817bp	LC472480.1	*Leishmania braziliensis*	16-1MD-L287	Peru
817bp	LC472479.1	*Leishmania braziliensis*	16-1MD-L271	Peru
817bp	LC472478.1	*Leishmania braziliensis*	16-1MD-L282	Peru
817bp	LC472477.1	*Leishmania braziliensis*	16-1MD-L265	Peru
817bp	LC472476.1	*Leishmania braziliensis*	16-1MD-L283	Peru
817bp	LC472475.1	*Leishmania braziliensis*	16-1MD-L284	Peru
817bp	LC472474.1	*Leishmania braziliensis*	16-1MD-L275	Peru
817bp	LC472473.1	*Leishmania braziliensis*	16-1MD-L286	Peru
817bp	LC472472.1	*Leishmania braziliensis*	16-1MD-L260	Peru
817bp	LC472471.1	*Leishmania braziliensis*	16-1MD-L289	Peru
817bp	LC472470.1	*Leishmania braziliensis*	16-1MD-L249	Peru
817bp	LC472469.1	*Leishmania braziliensis*	16-1MD-L291	Peru
817bp	LC472468.1	*Leishmania braziliensis*	16-1MD-L293	Peru
817bp	LC472466.1	*Leishmania braziliensis*	16-1MD-L218	Peru
817bp	LC472465.1	*Leishmania braziliensis*	16-1MD-L208	Peru
817bp	LC472464.1	*Leishmania braziliensis*	16-1MD-L298	Peru
817bp	LC472463.1	*Leishmania braziliensis*	16-1MD-L294	Peru
817bp	LC472462.1	*Leishmania braziliensis*	16-1MD-L361	Peru
817bp	LC472461.1	*Leishmania braziliensis*	16-1MD-L302	Peru
817bp	LC472460.1	*Leishmania braziliensis*	16-1MD-L315	Peru
817bp	LC472459.1	*Leishmania braziliensis*	16-1MD-L308	Peru
817bp	LC472458.1	*Leishmania braziliensis*	16-1MD-L310	Peru
817bp	LC472457.1	*Leishmania braziliensis*	16-1MD-L311	Peru
817bp	LC472453.1	*Leishmania braziliensis*	16-1PI-E271	Peru
817bp	LC472444.1	*Leishmania braziliensis*	16-1CU-SF33	Peru

Length	GenBank accession number	*Leishmania* specie	Isolate	Country of isolation
817bp	LC472445.1	*Leishmania braziliensis*	16-1AY-SF39	Peru
817bp	LC472441.1	*Leishmania braziliensis*	16-1AY-SF26	Peru
817bp	LC472440.1	*Leishmania braziliensis*	16-1AY-SF25	Peru
817bp	LC472428.1	*Leishmania braziliensis*	17-11MD-S6	Peru
817bp	LC472427.1	*Leishmania braziliensis*	17-11MD-S5	Peru
817bp	LC472426.1	*Leishmania braziliensis*	17-11MD-S3	Peru
817bp	LC472425.1	*Leishmania braziliensis*	17-11MD-S2	Peru
817bp	LC472424.1	*Leishmania braziliensis*	17-11PU1	Peru
817bp	LC472423.1	*Leishmania braziliensis*	17-11CU3	Peru
817bp	LC472422.1	*Leishmania braziliensis*	17-11HU2	Peru
817bp	LC472421.1	*Leishmania braziliensis*	17-11MD18	Peru
817bp	LC472420.1	*Leishmania braziliensis*	17-11MD16	Peru
817bp	LC472419.1	*Leishmania braziliensis*	17-11MD15	Peru
817bp	LC472418.1	*Leishmania braziliensis*	17-11MD14	Peru
817bp	LC472417.1	*Leishmania braziliensis*	17-11MD9	Peru
817bp	LC472416.1	*Leishmania braziliensis*	17-11MD8	Peru
817bp	LC472415.1	*Leishmania braziliensis*	17-11MD7	Peru
817bp	LC472414.1	*Leishmania braziliensis*	17-11MD6	Peru
817bp	LC472413.1	*Leishmania braziliensis*	17-11MD4	Peru
817bp	LC472412.1	*Leishmania braziliensis*	17-11MD3	Peru
817bp	LC472411.1	*Leishmania braziliensis*	17-11MD2	Peru
817bp	LC153269.1	*Leishmania braziliensis*	15-10DA2	Ecuador
817bp	LC153267.1*	*Leishmania braziliensis*	14-9PA-NM	Ecuador
817bp	LC153266.1	*Leishmania braziliensis*	14-9ZU-MA	Ecuador
817bp	LC153259.1	*Leishmania braziliensis*	14-2CBM9	Ecuador
426bp	LC153245.1	*Leishmania braziliensis*	12AR2	Ecuador
643bp	LC153240.1	*Leishmania braziliensis*	12.7-8EC619	Ecuador
817bp	LC153236.1	*Leishmania braziliensis*	13-9LA3	Ecuador
817bp	LC153230.1	*Leishmania braziliensis*	13-9CBM2	Ecuador
817bp	LC153232.1	*Leishmania braziliensis*	13-9L002	Ecuador
817bp	LC153228.1	*Leishmania braziliensis*	13-8EC6	Ecuador
817bp	LC153227.1	*Leishmania braziliensis*	13-8EC5	Ecuador
817bp	LC153222.1	*Leishmania braziliensis*	13-2EC11	Ecuador
817bp	LC153215.1	*Leishmania braziliensis*	13-2EC4	Ecuador
817bp	LC153196.1	*Leishmania braziliensis*	12PVM20	Ecuador
817bp	LC153185.1	*Leishmania braziliensis*	12PVM5	Ecuador
817bp	LC153167.1	*Leishmania braziliensis*	13CBM23	Ecuador
817bp	LC153160.1	*Leishmania braziliensis*	13CBM16	Ecuador
817bp	LC055634.1	*Leishmania braziliensis*	13AM4	Ecuador
817bp	LC055627.1	*Leishmania braziliensis*	15Or6	Ecuador
817bp	LC055622.1	*Leishmania braziliensis*	15Or1	Ecuador
817bp	AB558237.1	*Leishmania braziliensis*	Clone V-1	Venezuela
817bp	AB558236.1	*Leishmania braziliensis*	Clone V-5	Venezuela
817bp	AB558235.1	*Leishmania braziliensis*	Clone V-6	Venezuela
817bp	AB558234.1	*Leishmania braziliensis*	Clone V-7	Venezuela
817bp	AB558233.1	*Leishmania braziliensis*	Clone V-10	Venezuela
817bp	AB558232.1	*Leishmania braziliensis*	Clone V-12	Venezuela
816bp	AB558231.1	*Leishmania braziliensis*	Clone V-16	Venezuela
817bp	LC068832.1	*Leishmania braziliensis/ Leishmania peruviana*	-	Peru
817bp	AB433282.1*	*Leishmania peruviana*	LC39	-
908bp	MF278776.1*	*Leishmania peruviana*	MHOM/PE/87/PAB2880	Peru
899bp	MF278775.1*	*Leishmania peruviana*	MHOM/PE/84/LC26	Peru
817bp	LC472456.1	*Leishmania peruviana*	16-1PI-FYe	Peru
711bp	LC472876.1	*Leishmania peruviana*	12-2SI1	Peru
817bp	LC472455.1	*Leishmania peruviana*	16-1PI-E273	Peru
817bp	LC472454.1	*Leishmania peruviana*	16-1PI-E272	Peru
817bp	LC472451.1	*Leishmania peruviana*	16-1PI-E268	Peru
817bp	LC472450.1	*Leishmania peruviana*	16-1PI-E266	Peru
817bp	LC472449.1	*Leishmania peruviana*	16-1PI-E263	Peru
817bp	LC472448.1	*Leishmania peruviana*	16-1PI-E261	Peru

Length	GenBank accession number	*Leishmania* specie	Isolate	Country of isolation
817bp	LC472447.1	*Leishmania peruviana*	16-1PI-B43	Peru
817bp	LC472442.1	*Leishmania peruviana*	16-1AY-SF30	Peru
817bp	LC472439.1	*Leishmania peruviana*	12-2Sal16	Peru
817bp	LC472438.1	*Leishmania peruviana*	12-2Sal14	Peru
817bp	LC472438.1	*Leishmania peruviana*	12-2Sal14	Peru
817bp	LC472437.1	*Leishmania peruviana*	12-2Sal12	Peru
817bp	LC472436.1	*Leishmania peruviana*	12-2Sal11	Peru
817bp	LC472435.1	*Leishmania peruviana*	12-2Sal10	Peru
817bp	LC472434.1	*Leishmania peruviana*	12-2Sal9	Peru
817bp	LC472433.1	*Leishmania peruviana*	12-2Sal8	Peru
817bp	LC472432.1	*Leishmania peruviana*	12-2Sal7	Peru
817bp	LC472431.1	*Leishmania peruviana*	12-2Sal3	Peru
817bp	LC472430.1	*Leishmania peruviana*	12-2Sal2	Peru
817bp	LC472429.1	*Leishmania peruviana*	12-2Sal1	Peru
817bp	AB566382.1	*Leishmania peruviana*	09-per	Peru
817bp	AB566381.1*	*Leishmania peruviana*	08-per2	Peru
817bp	AB566380.1	*Leishmania peruviana*	08-per1	Peru
872bp	EF579905.1*	*Leishmania guyanensis*	LEM85	Peru
817bp	LC153252.1	*Leishmania guyanensis*	MAL11	Peru
817bp	LC153218.1	*Leishmania guyanensis*	13-2EC7	Ecuador
817bp	LC153199.1	*Leishmania guyanensis*	12PVM23	Ecuador
817bp	LC153193.1	*Leishmania guyanensis*	12PVM14	Ecuador
817bp	LC153186.1	*Leishmania guyanensis*	12PVM6	Ecuador
817bp	LC153180.1	*Leishmania guyanensis*	Que	Ecuador
817bp	LC153162.1	*Leishmania guyanensis*	13CBM18	Ecuador
817bp	LC472486.1	*Leishmania guyanensis*	16-12JU2	Ecuador
817bp	LC153277.1	*Leishmania guyanensis*	15-10VH111	Ecuador
817bp	LC153276.1	*Leishmania guyanensis*	15-10VH110	Ecuador
817bp	LC153275.1	*Leishmania guyanensis*	15-10VH108	Ecuador
817bp	LC153274.1	*Leishmania guyanensis*	15-10VH105	Ecuador
817bp	LC153273.1	*Leishmania guyanensis*	15-10VH100	Ecuador
817bp	LC153272.1	*Leishmania guyanensis*	15-10VH27	Ecuador
817bp	LC153265.1	*Leishmania guyanensis*	14-2CBM14	Ecuador
817bp	LC153264.1	*Leishmania guyanensis*	14-2CBM16	Ecuador
817bp	LC153263.1	*Leishmania guyanensis*	14-2CBM13	Ecuador
817bp	LC153262.1	*Leishmania guyanensis*	14-2CBM12	Ecuador
817bp	LC153261.1	*Leishmania guyanensis*	14-2CBM11	Ecuador
817bp	LC153260.1	*Leishmania guyanensis*	14-2CBM10	Ecuador
817bp	LC153258.1	*Leishmania guyanensis*	14-2CBM8	Ecuador
817bp	LC153254.1	*Leishmania guyanensis*	MAL13	Ecuador
817bp	LC153253.1	*Leishmania guyanensis*	MAL12	Ecuador
817bp	LC153251.1	*Leishmania guyanensis*	MAL10	Ecuador
817bp	LC153250.1	*Leishmania guyanensis*	MAL7	Ecuador
817bp	LC153249.1	*Leishmania guyanensis*	MAL5	Ecuador
817bp	LC153248.1	*Leishmania guyanensis*	MAL3	Ecuador
817bp	LC153247.1	*Leishmania guyanensis*	MAL1	Ecuador
817bp	LC153219.1	*Leishmania guyanensis*	13-2EC8	Ecuador
817bp	LC153217.1	*Leishmania guyanensis*	13-2EC6	Ecuador
817bp	LC153216.1	*Leishmania guyanensis*	13-2EC5	Ecuador
817bp	LC153214.1	*Leishmania guyanensis*	13-2EC1	Ecuador
817bp	LC153213.1	*Leishmania guyanensis*	12NA2	Ecuador
817bp	LC153212.1	*Leishmania guyanensis*	12NA1	Ecuador
817bp	LC153211.1	*Leishmania guyanensis*	12PEB2	Ecuador
817bp	LC153210.1*	*Leishmania guyanensis*	12LB9	Ecuador
817bp	LC153209.1	*Leishmania guyanensis*	12LB7	Ecuador
817bp	LC153208.1	*Leishmania guyanensis*	12LB6	Ecuador
817bp	LC153207.1	*Leishmania guyanensis*	12LB5	Ecuador
817bp	LC153206.1	*Leishmania guyanensis*	12LB1	Ecuador
817bp	LC153205.1	*Leishmania guyanensis*	12PVM32	Ecuador
817bp	LC153204.1	*Leishmania guyanensis*	12PVM31	Ecuador
817bp	LC153203.1	*Leishmania guyanensis*	12PVM30	Ecuador

Length	GenBank accession number	*Leishmania* specie	Isolate	Country of isolation
817bp	LC153202.1	*Leishmania guyanensis*	12PVM29	Ecuador
817bp	LC153201.1	*Leishmania guyanensis*	12PVM27	Ecuador
817bp	LC153200.1	*Leishmania guyanensis*	12PVM24	Ecuador
817bp	LC153198.1	*Leishmania guyanensis*	12PVM22	Ecuador
817bp	LC153198.1	*Leishmania guyanensis*	12PVM22	Ecuador
817bp	LC153197.1	*Leishmania guyanensis*	12PVM21	Ecuador
817bp	LC153195.1	*Leishmania guyanensis*	12PVM17	Ecuador
817bp	LC153192.1	*Leishmania guyanensis*	12PVM13	Ecuador
817bp	LC153191.1	*Leishmania guyanensis*	12PVM12	Ecuador
817bp	LC153190.1	*Leishmania guyanensis*	12PVM11	Ecuador
817bp	LC153189.1	*Leishmania guyanensis*	12PVM10	Ecuador
817bp	LC153188.1	*Leishmania guyanensis*	12PVM9	Ecuador
817bp	LC153187.1	*Leishmania guyanensis*	12PVM8	Ecuador
817bp	LC153184.1	*Leishmania guyanensis*	12PVM4	Ecuador
817bp	LC153183.1	*Leishmania guyanensis*	12PVM3	Ecuador
817bp	LC153181.1	*Leishmania guyanensis*	12PVM1	Ecuador
817bp	LC153165.1	*Leishmania guyanensis*	13CBM21	Ecuador
817bp	LC153164.1	*Leishmania guyanensis*	13CBM20	Ecuador
817bp	LC153163.1	*Leishmania guyanensis*	13CBM19	Ecuador
817bp	LC153161.1	*Leishmania guyanensis*	13CBM17	Ecuador
817bp	LC055621.1	*Leishmania guyanensis*	15VH4	Ecuador
817bp	LC055620.1	*Leishmania guyanensis*	15VH3	Ecuador
817bp	LC055619.1	*Leishmania guyanensis*	15VH2	Ecuador
817bp	LC055618.1	*Leishmania guyanensis*	15VH1	Ecuador
817bp	LC055638.1	*Leishmania guyanensis*	13AM11	Ecuador
817bp	LC055637.1*	*Leishmania guyanensis*	13AM8	Ecuador
817bp	LC055636.1	*Leishmania guyanensis*	13AM7	Ecuador
817bp	LC055631.1	*Leishmania guyanensis*	13-8EC11	Ecuador
817bp	LC055630.1	*Leishmania guyanensis*	13-8EC10	Ecuador
817bp	LC055628.1	*Leishmania guyanensis*	15Or7	Ecuador
817bp	LC055626.1	*Leishmania guyanensis*	15Or5	Ecuador
817bp	LC055625.1	*Leishmania guyanensis*	15Or4	Ecuador
817bp	LC055624.1	*Leishmania guyanensis*	15Or3	Ecuador
817bp	LC055623.1*	*Leishmania guyanensis*	15Or2	Ecuador
817bp	LC055617.1	*Leishmania guyanensis*	15Ca3	Ecuador
817bp	LC055616.1	*Leishmania guyanensis*	15Ca2	Ecuador
872bp	EF579901.1*	*Leishmania panamensis*	LEM702	Panama
509bp	EU499931.1	*Leishmania panamensis*	Lpasam1	Colombia
509bp	EU499924.1	*Leishmania panamensis*	Lpacol	Colombia
817bp	LC472482.1	*Leishmania lainsoni*	16-1MD87	Peru
714bp	LC472848.1	*Leishmania lainsoni*	12-2San4	Peru
713bp	LC472849.1	*Leishmania lainsoni*	12-2San5	Peru
713bp	LC472850.1	*Leishmania lainsoni*	12-2San6	Peru
713bp	LC472851.1	*Leishmania lainsoni*	12-2San7	Peru
713bp	LC472852.1	*Leishmania lainsoni*	12-2San10	Peru
715bp	LC472853.1	*Leishmania lainsoni*	12-2PP1	Peru
714bp	LC472856.1	*Leishmania lainsoni*	12-2PP4	Peru
714bp	LC472857.1	*Leishmania lainsoni*	12-2PP5	Peru
716bp	LC472858.1	*Leishmania lainsoni*	12-2PP6	Peru
713bp	LC472859.1	*Leishmania lainsoni*	12-2PP7	Peru
726bp	LC472867.1	*Leishmania lainsoni*	12-2AA1	Peru
711bp	LC472877.1	*Leishmania lainsoni*	12-2MS1	Peru
703bp	LC472878.1	*Leishmania lainsoni*	12-2MS2	Peru
695bp	LC472879.1	*Leishmania lainsoni*	12-2MS3	Peru
817bp	AB433280.1	*Leishmania lainsoni*	MHOM/BR/81/M6426	Brazil
817bp	LC472467.1	*Leishmania lainsoni*	16-1MD-L296	Peru
817bp	LC472446.1	*Leishmania lainsoni*	16-1CU-LRRA759	Peru
817bp	LC153271.1*	*Leishmania lainsoni*	15-10CA2	Ecuador
817bp	LC153270.1*	*Leishmania lainsoni*	15-10CA1	Ecuador
817bp	LC055632.1	*Leishmania lainsoni*	13-8EC14	Ecuador
817bp	LC055629.1	*Leishmania lainsoni*	13-8EC7	Ecuador

Length	GenBank accession number	*Leishmania* specie	Isolate	Country of isolation
817bp	AB433279.1	*Leishmania naiffi*	MDAS/BR/79/M5533	Brazil
817bp	LC153257.1*	*Leishmania naiffi*	2CBM5	Ecuador
817bp	LC153256.1*	*Leishmania naiffi*	2CBM4	Ecuador
817bp	LC153224.1*	*Leishmania naiffi*	8EC2	Ecuador
817bp	LC153223.1*	*Leishmania naiffi*	8EC1	Ecuador
817bp	AB433281.1	*Leishmania shawi*	MHOM/BR/79/M15065	Brazil
872bp	EF579915.1	*Leishmania mexicana*	MHOM/MX/93/CRE47	Mexico
872bp	EF579906.1	*Leishmania mexicana*	LEM695	Belize
817bp	LC416876.1	*Leishmania mexicana*	18LC0002S	Venezuela
817bp	LC416875.1	*Leishmania mexicana*	18Ven_Fel14	Venezuela
817bp	LC416874.1	*Leishmania mexicana*	18Ven_Fel13L	Venezuela
817bp	LC416873.1	*Leishmania mexicana*	18Ven_Fel13R	Venezuela
817bp	LC416872.1	*Leishmania mexicana*	18Ven_Fel11	Venezuela
817bp	LC416871.1	*Leishmania mexicana*	18Ven_Fel10	Venezuela
817bp	LC416870.1	*Leishmania mexicana*	18Ven_Fel9	Venezuela
817bp	LC153244.1	*Leishmania mexicana*	12-7HU1	Ecuador
817bp	LC386854.1	*Leishmania mexicana*	17Ven_Can2	Venezuela
817bp	LC386853.1	*Leishmania mexicana*	17Ven_Fel8	Venezuela
817bp	LC386852.1	*Leishmania mexicana*	17Ven_Fel7	Venezuela
817bp	LC386851.1	*Leishmania mexicana*	17Ven_Fel6	Venezuela
817bp	LC386850.1	*Leishmania mexicana*	17Ven_Fel5	Venezuela
817bp	LC386849.1	*Leishmania mexicana*	17Ven_Fel4	Venezuela
817bp	AB558230.1	*Leishmania mexicana*	V-2	Venezuela
817bp	AB558229.1	*Leishmania mexicana*	V-3	Venezuela
817bp	AB558228.1	*Leishmania mexicana*	V-4	Venezuela
817bp	AB558227.1	*Leishmania mexicana*	V-9	Venezuela
817bp	AB558226.1	*Leishmania mexicana*	V-11	Venezuela
817bp	AB558225.1	*Leishmania mexicana*	V-13	Venezuela
817bp	AB558224.1	*Leishmania mexicana*	V-14	Venezuela
817bp	AB558223.1	*Leishmania mexicana*	V-15	Venezuela
817bp	AB558222.1	*Leishmania mexicana*	V-17	Venezuela
817bp	AB558221.1	*Leishmania mexicana*	Hu2-5F	Venezuela
817bp	AB558220.1	*Leishmania mexicana*	Hu1-11B	Venezuela
817bp	AB558219.1	*Leishmania mexicana*	Hu1-7G	Venezuela
817bp	AB558218.1	*Leishmania mexicana*	Huigra1	Venezuela
817bp	AB558217.1	*Leishmania mexicana*	DCL An	Venezuela
816bp	AB847156.1	*Leishmania mexicana*	Cha-2G	Venezuela
817bp	AB847155.1	*Leishmania mexicana*	Cha-7F	Ecuador
817bp	AB847154.1	*Leishmania mexicana*	Chan-3A	Ecuador
817bp	AB847153.1	*Leishmania mexicana*	Ala-16D	Ecuador
817bp	AB847152.1	*Leishmania mexicana*	Ala-10F	Ecuador
817bp	AB847151.1	*Leishmania mexicana*	Ala-6G	Ecuador
816bp	AB847150.1	*Leishmania mexicana*	Ala-3G	Ecuador
816bp	AB847149.1	*Leishmania mexicana*	Ala-3A	Ecuador
872bp	EF579909.1	*Leishmania amazonensis*	LMAMPRO/BR/72/M1841	Brazil
872bp	EF579902.1	*Leishmania amazonensis*	LEM690<br>MHOM/BR/73/M2269	Brazil
730bp	MF344895.1	*Leishmania amazonensis*	EM_246	Brazil
730bp	MF344894.1	*Leishmania amazonensis*	EM_400	Brazil
730bp	MF344893.1	*Leishmania amazonensis*	EM_394	Brazil
730bp	MF344892.1	*Leishmania amazonensis*	EM_274	Brazil
730bp	MF344891.1	*Leishmania amazonensis*	EM_273	Brazil
730bp	MF344890.1	*Leishmania amazonensis*	EM_272	Brazil
730bp	MF344889.1	*Leishmania amazonensis*	EM_269	Brazil
730bp	MF344888.1	*Leishmania amazonensis*	EM_266	Brazil
730bp	MF344887.1	*Leishmania amazonensis*	EM_253	Brazil
730bp	MF344886.1	*Leishmania amazonensis*	EM_176	Brazil
730bp	MF344885.1	*Leishmania amazonensis*	EM_130	Brazil
730bp	MF344884.1	*Leishmania amazonensis*	EM_087	Brazil
730bp	MF344883.1	*Leishmania amazonensis*	EM_053	Brazil
730bp	MF344882.1	*Leishmania amazonensis*	EM_042	Brazil

Length	GenBank accession number	*Leishmania* specie	Isolate	Country of isolation
817bp	LC472443.1	*Leishmania amazonensis*	16-1JU-LRRA488	Peru
839bp	LC089019.1	*Leishmania infantum*	Leish38_Cytb_EX	Spain
872bp	EF579913.1	*Leishmania infantum*	MCAN/GR/94/CRE69	Greece
872bp	EF579895.1	*Leishmania infantum*	MHOM/TN/80/IPT1	-
800bp	KX061917.1	*Leishmania infantum*	MHOM/CN/80/801	China
797bp	KX061912.1	*Leishmania infantum*	MCAN/CN/90/WenChuan	China
825bp	KX061911.1	*Leishmania infantum*	MCAN/CN/08/Cy	China
730bp	MF344874.1	*Leishmania infantum*	EM_382	Brazil
730bp	MF344873.1	*Leishmania infantum*	EM_267	Brazil
730bp	MF344872.1	*Leishmania infantum*	EM_395	Brazil
730bp	MF344871.1	*Leishmania infantum*	EM_387	Brazil
730bp	MF344870.1	*Leishmania infantum*	EM_378	Brazil
730bp	MF344869.1	*Leishmania infantum*	EM_264	Brazil
730bp	MF344868.1	*Leishmania infantum*	EM_361	Brazil
730bp	MF344867.1	*Leishmania infantum*	EM_177	Brazil
730bp	MF344866.1	*Leishmania infantum*	EM_243	Brazil
730bp	MF344865.1	*Leishmania infantum*	EM_169	Brazil
730bp	MF344864.1	*Leishmania infantum*	EM_143	Brazil
730bp	MF344863.1	*Leishmania infantum*	EM_142	Brazil
730bp	MF344862.1	*Leishmania infantum*	EM_116	Brazil
730bp	MF344861.1	*Leishmania infantum*	EM_101	Brazil
730bp	MF344860.1	*Leishmania infantum*	EM_005	Brazil
847bp	LC089019.1	*Leishmania infantum*	Leish38_Cytb_EX	Spain
872bp	EF579911.1	*Leishmania donovani*	MHOM/IN/80/DD8	India
872bp	EF579896.1	*Leishmania donovani*	MHOM/IN/80/DD8	India
833bp	KX061916.1	*Leishmania donovani*	MHOM/CN/86/SC6	China
807bp	KX061915.1	*Leishmania donovani*	IMJW/CN/92/KXG-927	China
825bp	KX061906.1	*Leishmania donovani*	MHOM/CN/93/KXG-XU	China
828bp	KX061905.1	*Leishmania donovani*	IMJW/CN/87/KXG-65	China
811bp	KX061904.1	*Leishmania donovani*	IMJW/CN/91/KXG-918	China
872bp	EF579897.1	*Leishmania donovani*	MHOM/BR/74/PP75a	Brazil
702bp	AB725910.1	*Leishmania donovani*	D81	Bangladesh
795bp	KX061908.1	*Leishmania donovani*	MHOM/CN/94/KXG-LIU	China
767bp	KX061907.1	*Leishmania donovani*	MHOM/CN/90/9044	China

*: *Leishmania* reference sequences used for the phylogenetic analysis.

TABLE II

Singletons and parsimony-informative sites found in cytochrome b (cytb) sequences analysed in this study

	Nucleotide sites																						
Leishmania species	44	47	48	62	92	95	98	107	116	119	120	122	140	146	152	170	179	182	194	197	198	200	221
L. panamensis	G	A	A	C*	A	A	A	T	A	A	C	A	T	T	A	A	T	T	G	A	A	A	A
L. guyanensis	G	A	A	T	A	A	A	T	A	A	Y	A	T	T	A	A	T	T	G	A	A	A	A
L. braziliensis	G	A	A	T	A	A	A	T	A	A	T	A	T	T	A	G	T	T	K	A	A	A	A
*L. peruviana***	-	-	-	-	A	A	A	T	A	A	T	A	A	T	A	G	T	T	G	A	A	A	A
*L. shawi***	G	A	A-	T	A	A	A	T	A	A	C	A	T	T	A	A	T	T	G	A	A	A	A
*L. naiffi***	-	-	-	-	A	A	A	T	A	A	C	A	T	T	A	A	T	T	G	A	A	A	A
L. lainsoni	-	-	-	-	A	W	A	T	A	A	C	A	A	T	A	A	T	T	G	A	A	A	A
L. mexicana	A	A	G	T	A	T	T	A	A	A	C	T	A	A	G	A	T	T	A	T*	A	A	A
L. amazonensis	A	A	G	T	A	T	T	G	A	A	C	T	A	A	G	A	T	T	W	A	C	A	T
L. infantum	A	T	G	T	G*	A	A	G	G*	G*	T	A	A	A	A	A	C*	C*	A	A	C	A	T

	Nucleotide sites																						
	224	228	236	240	242	246	248	260	269	278	281	287	290	302	306	308	309	318	323	329	338	339	344
L. panamensis	A	C	A	C	T	G	T	A	A	A	A	T	T	T	A	T	A	A	T	A	A	A	A
L. guyanensis	A	C	A	C	K	G	T	A	A	A	A	T	T	T	A	T	A	A	T	A	A	A	A
L. braziliensis	A	C	A	C	T	G	T	A	A	A	A	T	T	T	A	T	A	A	T	A	A	A	A
L. peruviana	A	C	A	C	T	G	T	A	A	A	A	T	T	T	A	T	A	A	T	A	A	A	A
L. shawi	A	T	A	C	T	G	T	G	A	A	A	T	T	T	A	T	A	A	T	A	A	A	A
L. naiffi	A	T	A	C	T	G	W	A	A	A	A	T	T	T	G	T	A	A	T	A	A	G	A
L. lainsoni	A	T	A	C	T	G	Y	A	A	A	A	T	T	T	A	T	A	A	T	A	A	G	A
L. mexicana	T	T	C	T	A	A	T	G	R	Y	G	T	T	G	G	A	G	G	A	A	C	A	C
L. amazonensis	T	T	C	T	A	A	W	G	A	T	A	C*	T	G	G	A	G	G	A	T*	T	A	T
L. infantum	A	T	C	C	T	G	C	G	A	A	G	T	A*	A	G	S	G	G	A	A	A	A	A

	Nucleotide sites																						
	362	363	365	368	374	383	392	398	401	414	419	422	425	428	431	434	437	438	439	443	444	446	458
L. panamensis	A	C*	G	G*	A	G	A	A	T	T	T	T	G	A	G*	T	T	G	T	A	T	A	T
L. guyanensis	A	T	G	A	A	G	A	A	T	C*	T	T	G	A	T	T	T	G	T	A	C	A	T
L. braziliensis	A	T	G	A	A	G	A	A	T	T	T	T	G	A	T	T	T	G	T	A	Y	A	T
L. peruviana	A	T	G	A	A	G	A	A	T	T	T	T	G	A	T	T	T	G	T	A	T	A	T
L. shawi	A	T	G	A	A	G	A	A	T	T	T	T	G	A	T	T	T	G	T	A	T	A	T
L. naiffi	A	T	G	A	A	G	A	A	T	T	T	T	A	A	T	T	T	G	T	G*	T	A	T
L. lainsoni	A	T	G	A	A	A	A	A	T	T	T	T	A	A	T	T	T	G	T	A	C	A	T
L. mexicana	T	T	A	A	A	T	T	T	A	T	A	A	A	T	T	A	Y	A	C	A	C	T	A
L. amazonensis	T	T	A	A	A	T	C	T	A	T	A	A	A	T	T	A	C	A	C	A	C	T	A
L. infantum	A	T	A	A	G*	G	T	A	A	T	A	C	C	A	T	T	T	A	C	A	C	T	T

	Nucleotide sites																						
	464	476	485	488	489	492	494	506	519	521	524	527	528	530	534	537	539	540	542	545	563	584	590
L. panamensis	A	A	T	T	T	C	T	T	C	A	T	T	A	T	T	A	A	A	A	T	T	T	T
L. guyanensis	A	A	T	T	T	C	T	T	C	A	T	T	A	T	T	A	A	A	A	T	T	T	T
L. braziliensis	A	A	T	T	T	C	T	T	C	A	T	T	A	T	T	A	A	A	A	T	T	T	T
L. peruviana	A	A	T	T	T	C	T	T	C	A	T	T	A	T	T	A	A	A	A	T	T	T	T
L. shawi	A	A	T	T	T	C	T	T	C	A	T	T	A	T	T	A	A	A	A	T	T	T	T
L. naiffi	A	A	T	T	T	C	T	T	T	A	T	T	A	T	T	A	A	A	A	T	T	T	T
L. lainsoni	A	A	C*	T	T	C	T	T	T	A	T	T	A	T	T	A	A	A	A	T	C*	T	T
L. mexicana	T	T	T	A	C*	C	T	T	T	A	T	T	G	A	A	T	A	G	G	M	T	A	T
L. amazonensis	T	T	T	A	T	C	T	T	T	G	T	T	G	A	A	T	A	G	T	A	T	A	T
L. infantum	T	T	T	A	T	T*	A*	A*	T	A	A*	C*	G	A	A	C	T*	G	A	A	T	A	G*

	Nucleotide sites																						
	614	617	620	623	624	641	651	668	680	685	687	689	690	692	701	704	707	722	731	734	737	740	743
L. panamensis	T	T	G	A	T	A	A	G*	A	T	G	A	G	G	A	T	A	T	G	G	A	A	A
L. guyanensis	T	T	R	A	C	A	A	A	A	T	R	A	G	G	A	T	A	T	G	G	A	A	A
L. braziliensis	T	T	G	G	C	A	A	A	A	T	G	A	G	G	A	T	A	T	G	G	A	A	A
L. peruviana	T	T	G	G	C	A	A	A	A	T	G	A	G	G	A	T	A	T	G	G	A	A	A
L. shawi	T	T	G	A	T	A	A	A	A	T	G	A	G	G	A	T	A	T	G	A*	A	A	A
L. naiffi	C*	T	G	A	T	A	A	A	A	T	G	A	G	G	A	T	A	T	G	G	A	A	A
L. lainsoni	T	C*	G	T	T	A	A	A	G	T	G	A	G	G	A	T	A	T	G	G	A	A	A
L. mexicana	T	T	A	T	T	G	A	A	A	A	G	W	A	A	T	T	T	T	A	G	T	G	T
L. amazonensis	T	T	A	T	T	G	A	A	A	A	G	A	A	A	T	C*	T	T	A	G	T	G	T
L. infantum	T	T	A	T	T	A	C*	A	G	T	G	G	A	T	T	T	A	A*	A	G	A	A	A

	Nucleotide sites																						
	746	749	752	764	773	777	779	782	788	800	806	818	827	833	846	848	860	861	866	875	881	896	911
L. panamensis	A	A	C	T	A	T	A	T	G	T	T	A	T	A	T	A	A	C	A	G	A	T	T
L. guyanensis	A	A	C	T	A	T	A	T	G	T	T	A	T	A	T	A	A	Y	A	G	A	T	T
L. braziliensis	A	A	C	T	A	T	A	T	G	T	T	A	T	A	T	A	A	Y	A	G	A	T	T
L. peruviana	A	A	C	T	A	T	A	T	G	T	T	A	T	A	T	A	A	T	A	G	A	-	-
L. shawi	A	A	C	T	A	T	A	T	G	T	T	A	T	A	T	A	A	C	A	G	A	T	T
L. naiffi	A	G	T	T	A	T	A	T	A	T	T	A	T	A	T	A	A	T	A	G	A	T	T
L. lainsoni	A	A	T	T	A	T	A	T	A	T	T	A	T	A	T	A	A	Y	A	G	A	-	-
L. mexicana	T	A	T	C	A	C	G	A	G	T	C	A	A	A	C	A	T	T	G	A	G	T	A
L. amazonensis	T	G	T	A	A	T	G	T	G	T	C	A	A	A	T	G*	T	T	G	A	G	T	A
L. infantum	A	T	T	A	G*	C	C	A	A	C*	T	G*	T	G*	C	A	T	T	G	A	G	C*	T

	Nucleotide sites						
	918	926	929	932	938	941	944
L. panamensis	C	G	G	A	T	T	T
L. guyanensis	C	G	G	A	T	T	T
L. braziliensis	C	G	G	A	T	T	T
L. peruviana	-	-	-	-	-	-	-
L. shawi	C	G	A	A	T	T	T
L. naiffi	-	-	-	-	-	-	-
L. lainsoni	-	-	-	-	-	-	-
L. mexicana	A	A	A	A	T	A	T
L. amazonensis	A	A	A	A	T	A	T
L. infantum	A	A	G	G*	C*	T	C*

*: singletons; **: only partial cytb sequences were obtained for these *Leishmania* species.
